# Littoral cell angioma of the spleen: A study of 10 cases case series and literature review

**DOI:** 10.1097/MD.0000000000037550

**Published:** 2024-03-29

**Authors:** Huaping Shen, Yingjie Zhu, Jiajie Zhong, Yang Shen, Yang Huang, Pengtao Song, Jian He, Shiyu Zhou, Xiaochang Wu

**Affiliations:** a Department of General Surgery, Huzhou Central Hospital, Affiliated Central Hospital of Huzhou University, Huzhou, China; b Fifth School of Clinical Medicine of Zhejiang Chinese Medical University, Huzhou, China; c Department of Pathology, Huzhou Central Hospital, Affiliated Central Hospital of Huzhou University, Huzhou, China; d Department of Radiology, Huzhou Central Hospital, Affiliated Central Hospital of Huzhou University, Huzhou, China.

**Keywords:** diagnosis, littoral cell angioma, splenectomy, splenic tumor

## Abstract

**Background:**

Current study aimed to investigate the clinical characterization, differential diagnosis, and treatment of splenic littoral cell angioma (LCA).

**Methods:**

A retrospective analysis was performed for 10 LCA cases admitted to Huzhou Central Hospital from 2007 to 2023, for clinical manifestations, hematological tests, imaging features, pathological features, treatment methods, and prognosis along with the relevant literature was also reviewed.

**Results:**

During examinations, no specific clinical manifestations and hematological abnormalities were seen in all 10 cases of LCA. Imaging observations depicted single or even multiple spherical lesions in the spleen. Plains shown by computed tomography (CT) were found somewhat equal or slightly lower in density. On the other hand, magnetic resonance imaging (MRI) plain scans viz. T1 weighted image showed equal low and mixed signals while T2-weighted showed high and low mixed signals. Moreover, punctate low signals could be seen in high signals named “freckle sign” in MRI scans. On contrast-enhanced CT scans, the enhancement of the lesions was not obvious in the arterial phase, and some of the lesions showed edged ring-like enhancements and “filling lake” progressive enhancement during the venous phase and delayed phase. In multiple lesions, the number of enhanced scan lesions showed a variable changing pattern “less-more-less.” MRI-enhanced scan showed the characteristics of “fast in and slow out.” Microscopic examinations identified tumor tissue actually composed of sinus-like lacunae that anastomosed with each other in the form of a network. Furthermore, cystic expansion and pseudopapillary protrusions were also seen in the dilated sinus cavity which was lined with single-layer endothelial cells having conspicuous cytoplasmic hemosiderin. High immunophenotypic expressions of vascular endothelial cell phenotype (CD31, CD34, FVIII) and tissue cell phenotype (CD68) were also seen. Total and partial splenectomy were performed in 8 and 2 patients, respectively, and follow-up examinations showed survival in all patients with no recurrence.

**Conclusion:**

LCA is a rare splenic benign lesion with atypical clinical manifestations. CT and MRI imaging are important tools in preoperative diagnosis based on pathomorphological and immunohistochemical examinations. Splenectomy is a superior therapeutic choice with significant impacts and prognosis.

## 1. Introduction

Splenic littoral cell angioma (LCA) is a benign vascular tumor originating from the splenic endothelial reticular system. It is among the rare tumors occuring in the spleen. The disease was first reported by Falk and colleagues in the early 90s, according to the research group the disease prognosis is much better.^[1]^ Since LCA has no typical clinical signs in imaging examinations, it is quite difficult to differentiate it from other benign or malignant splenic tumors before surgery. Primarily the absolute diagnosis solely depends on pathological examinations. At present, clinicians have little understanding of LCA, which can easily mislead in its proper diagnosis or completely miss the detection of the disease. Herein, the study aimed to evaluate the 10 cases of LCA that remained admitted to Huzhou Central Hospital from 2007 to 2023 for comprehensive retrospective analyses to explore its clinical manifestations, imaging and pathological features, treatment methods, and prognosis in order to clearly define clinical understanding of this disease.

## 2. Materials and methods

### 2.1. Clinical data

The clinical data of 10 patients with LCA diagnosed by pathology in Huzhou Central Hospital from 2007 to 2023 was retrospectively analyzed. The study was comprised of 4 male and 6 female patients aged 24 to 59 years, with an average age of 44 ± 10.7 years. The written consent from the patients was done, and the study was performed according to the ethical regulations set by the Huzhou Central Hospital, China. The clinical characteristics of the patients were analyzed to identify the initial symptoms, abdominal signs, auxiliary examinations, pathological features, and treatment regimen.

### 2.2. Examination methods

Among the 10 patients, 8 patients underwent abdominal color Doppler ultrasound, 4 patients underwent abdominal computed tomography (CT) enhanced scan, 4 patients underwent abdominal magnetic resonance imaging (MRI) enhanced scan, while 3 patients underwent only abdominal CT plain scan.

Ultrasound examination was performed by specialized sonographers being supervised by physicians holding at least 3 years of technical experience. Color Doppler ultrasound was conducted utilizing the Philips IU22 diagnostic ultrasound instrument.

TOSHIBA Aquilion 16-row spiral CT machine was used to perform upper abdominal plain scan and 3-stage enhanced scans. Scanning parameters includes tube current 150mAs, tube voltage 120Kvp, collimation 0.5 mm × 16/1 mm × 16, and matrix 512 × 512. Omnipaque 80 to 100 mL was injected through the anterior cubital vein at a rate of 3 mL/s. The 3 phases of enhancement were scanned at 25 to 30 seconds (arterial phase), 65 to 80 seconds (portal phase), and 120 to 150 seconds (delayed phase) after the start of contrast injection.

MRI examination used GE signa excite 1.5T HD superconducting magnetic resonance instrument and 8-channel body phased array coil. Scanning sequence: Cross-sectional fat suppression GRE T1 weighted (T1WI); cross-sectional fat suppression FRFSE T2-weighted (T2WI), axial DWI scan using SE-EPI sequence, take the *b* value of 0 and 700 seconds/mm^2^. Liver acquisition with volume acceleration sequence of transverse fat suppression T1WI was used for enhancement. The contrast agent was Gd-DTPA, the dose was 0.2 mmol/kg, and the injection rate was 3.0 mL/s (intravenous injection through the antecubital vein). The arterial phase scan was performed 15 seconds after the start of contrast injection, the portal phase scan was performed 45 seconds after the start of contrast injection, and the delayed phase scan was performed in the 90 seconds.

### 2.3. Analysis of image and clinical features

Double-blind image reading, CT and MRI image analysis, and ultrasound exploration and diagnosis were performed by 2 radiologists with associate senior titles. The number, size, shape, boundary, and enhancement characteristics of tumors were mainly observed. The cases with different opinions were determined by relevant senior physicians.

### 2.4. Pathological specimens

The spleen specimens were initially fixed in 10% formaldehyde solution, dehydrated, sectioned in paraffin, and subjected to hematoxylin and eosin staining. Simultaneously, immunohistochemistry was carried out to label CD31, CD34, FVIII, CD68, and Ki-67. To confirm the diagnosis, the morphological characteristics and immunophenotype of the specimens were observed under a digital microscope equipped with high high-definition camera.

### 2.5. Statistical analysis

The various data for clinical characteristics of the patients including the initial symptoms, abdominal signs, auxiliary examinations, pathological features, and treatment methods were gone through the analysis using Microsoft Excel for descriptive statistics.

## 3. Results

### 3.1. General clinical data

Among the 10 patients with LCA, 5 patients were diagnosed due to the discovery of masses during physical examination, while the remaining 5 patients presented with symptoms of abdominal pain. One patient was complicated with gallstones, and another was complicated with a lung malignant tumor. All the patients had no unexplained fever, thrombocytopenic purpura, anemia, or any other blood system diseases. Five patients showed single while the rest of the 5 patients showed multiple lesions (Table 1).

**Table 1:** Summarized data of 10 clinical cases of littoral cell angioma.

Case	Age	Sex	Clinical manifestation	Physical examination		Assistant examination	Single/multiple tumors	Lesion size (cm)	Immunohistochemical					Treatment
				Abdominal tenderness	Splenomegaly				CD31	CD34	FVIII	CD68	Ki-67	
1	43	F	Abdominal pain (left upper)	No	No	Ultrasonography, CT plain scan	Multiple	N/A	N/A	N/A	N/A	N/A	N/A	Open splenectomy
2	39	M	Medical check	No	No	Ultrasonography, CT plain scan	Single	3.9	N/A	+	+	N/A	(±)	Open splenectomy
3	38	F	Abdominal pain (left upper)	No	No	Ultrasonography, CT+	Multiple	1.5	N/A	+	N/A	+	+	Open splenectomy
4	24	M	Abdominal pain (upper middle)	Yes	No	Ultrasonography, CT+	Single	5.0	N/A	−	N/A	+	+5%	Laparoscopic partial splenectomy
5	59	F	Abdominal pain (right upper)	No	Yes	CT plain scan, MR+	Multiple	N/A	+	+	+	+	+5%	Laparoscopic splenectomy
6	49	M	Abdominal pain (upper middle)	Yes	Yes	Ultrasonography,CT+, MR+	Multiple	2.2	+	−	N/A	N/A	+5%	Laparoscopic splenectomy
7	53	F	Medical check	No	No	Ultrasonography, CT+,	Multiple	1.5	+	−	+	+	+5%	Laparoscopic splenectomy
8	43	F	Medical check	No	No	MR+	Single	4.5	+	+	−	+	+2%	Laparoscopic splenectomy
9	33	F	Medical check	No	No	Ultrasonography,	Single	3.2	+	+	N/A	−	+3%	Laparoscopic splenectomy
10	59	M	Medical check	No	No	Ultrasonography, MR+	Single	4.6	+	+	+	+	+3%	Laparoscopic partial splenectomy

CT = computed tomography.

### 3.2. CT scan

**Figure 1.**
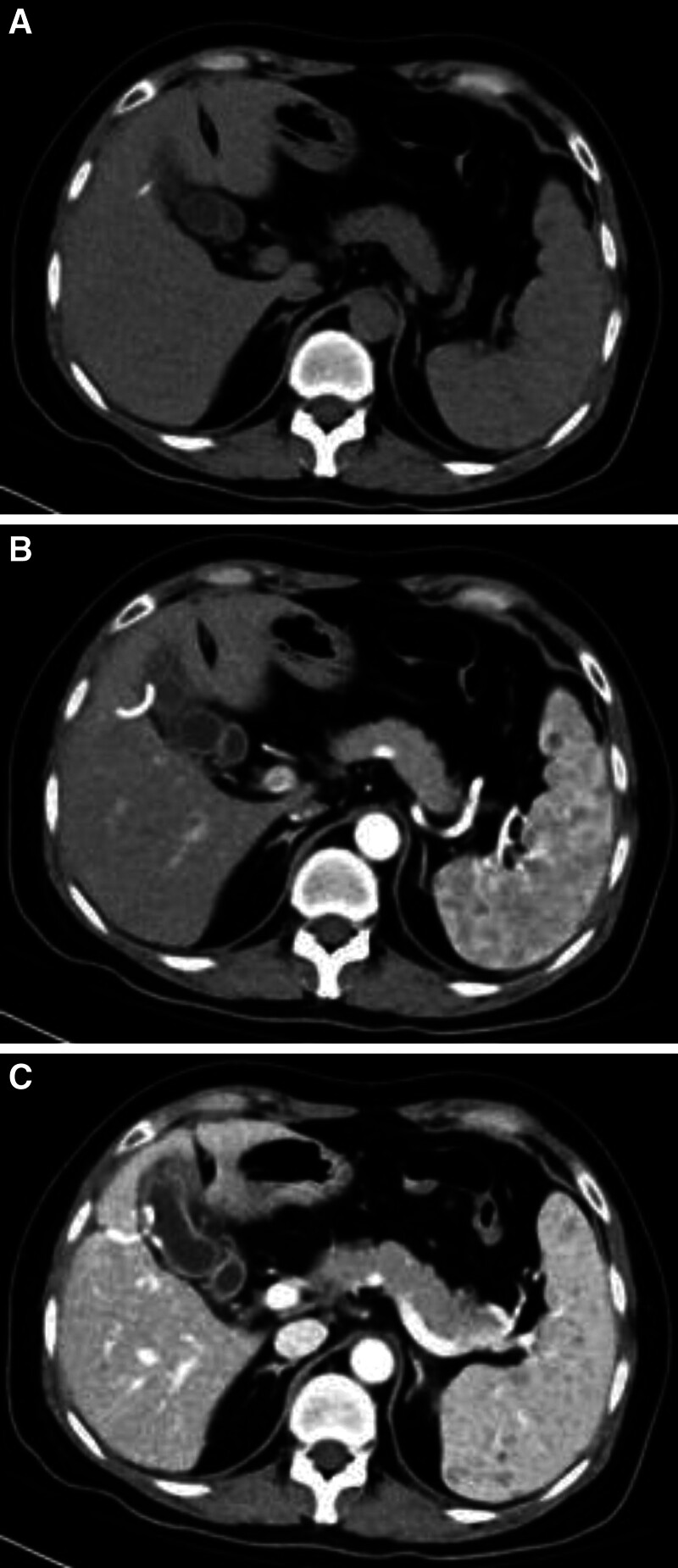
(A) The LCA mass exhibited equal or slightly lower density (compared with the spleen). (B) The enhancement of the lesion was not obvious in the arterial phase of the enhanced scan, and some of the lesion edge was ring-like enhancement. (C) The venous phase and delayed phase showed “lake-filling” progressive enhancement, and the number of enhanced scanning lesions showed a dynamic change process of “less-more-less.” LCA = littoral cell angioma.

In 4 patients, an abdominal enhanced CT examination was performed, while 3 patients were examined for an abdominal plain CT scan. The plain CT scan revealed that the LCA mass exhibited equal or slightly lower density (compared to the spleen) (Fig. 1A). During the arterial phase of the enhanced scan, the lesion showed minimal enhancement with partial ring-like enhancement at the edges (Fig. 1B). In the venous and delayed phases, progressive enhancement resembling a “filling lake” pattern was observed in the lesion. For patients with multiple lesions, the number of enhanced scan lesions showed a dynamic change process of “less-more-less” (Fig. 1C).

### 3.3. MRI examination

**Figure 2.**
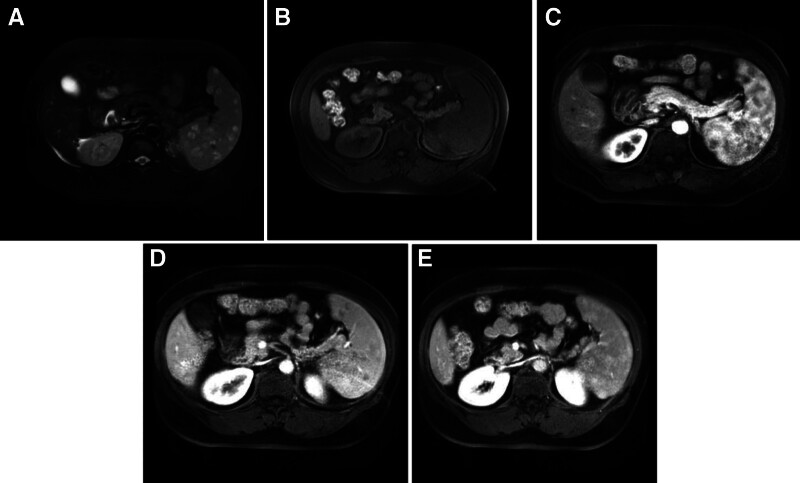
(A) T2WI showed lesions with a mixed high and low signal intensity. Within the high signal area, punctate low signals were observed, resembling a “freckle-like” appearance. (B) T1WI revealed iso-low hypointense signals with mixed intensity. (C) MRI enhanced the arterial phase with mild enhancement around the lesion. (D and E) MRI enhanced the portal phase and balance phase, with the further slow centripetal enhancement of the lesion. MRI = magnetic resonance imaging, T1WI = T1 weighted, T2WI = T2-weighted.

Four patients underwent an MRI examination, where T2WI showed lesions with mixed high and low signal intensity. Within the high signal area, punctate low signals were observed, resembling a “freckle-like” appearance (Fig. 2A). The MRI T1WI revealed iso-low hypointense signals with mixed intensity (Fig. 2B). The lesions did not enhance in the arterial phase but enhanced in the delayed phase. The contrast-enhanced scans exhibited a “fast-in, slow-out” pattern (Fig. 2C–E).

### 3.4. Pathological results

Ten patients were diagnosed as LCA by pathology, all of which were found benign lesions. Among them, 2 patients had splenomegaly, while the spleen volume was normal rest of the 8 patients. Multiple or solitary nodules can be observed on the surface or sections of the spleen, out of which 5 patients showed multiple nodules and the rest presented single nodules each. There was normal splenic tissue between the nodules, the sections of the nodules were spongy or either cystic. The colors, were mostly grayish red, and the boundaries at the surrounding splenic tissue were still clear.

**Figure 3.**
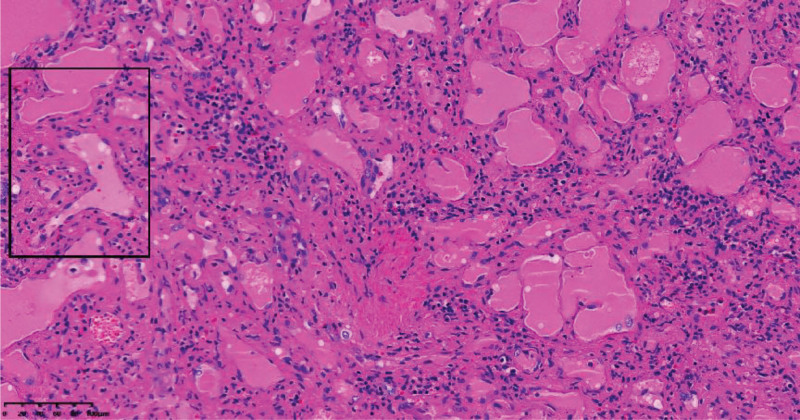
Photomicrograph of the histologic specimen (HE staining, ×200) shows that Tumors are composed of sinusoid-like cavities of varying sizes, some of which are cystic in expansion, with a single layer of endothelial cells lining the walls, and the cytoplasm contained hemosiderin was visible. HE staining = hematoxylin-eosin staining.

Microscopic examinations showed that the tumor tissue was composed of sinus-like lacunae that anastomosed with each other and formed a network. Some of them had cystic expansion. Pseudopapillary protrusions were visible in the dilated sinuses, the wall of the cavity was lined with a single layer of endothelial cells whose cytoplasm contained conspicuous hemosiderin, the nuclei were oval and the mitotic images were rare (Fig. 3).

**Figure 4.**
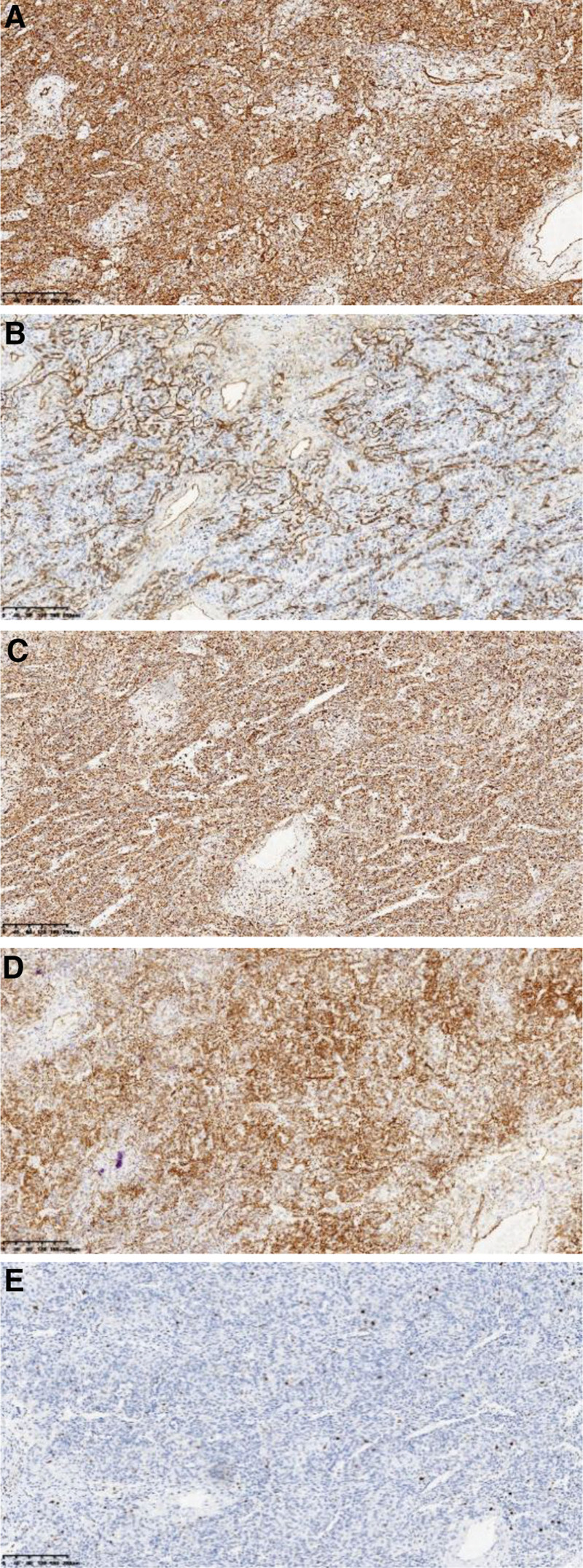
(A) CD31 was strong positively expressed. (B) CD34 was strong positively expressed. (C) CD68 was strong positively expressed. (D) FVIII was strong positively expressed. (E) Ki-67 proliferation index was about 5%.

In about 9 patients of LCA, the immunohistochemical (IHC) staining showed vascular markers viz. CD31 positive in 6 cases (66.7%, 6/9), CD34 positive in 6 cases (66.7%, 6/9), FVIII positive in 4 cases (44.4%, 4/9). Tissue cell markers: 6 cases (66.7%, 6/9) of CD68 positive, while epithelial markers were all negative, the Ki-67 proliferation index was 2% to 5% (Fig. 4A–E).

## 4. Discussion

### 4.1. Clinical features

Splenic LCA mainly originates from the littoral cells of the red pulp of the spleen. The pathogenesis and biological behavior are not clear, and there are few related research reports. Although LCA is a benign tumor, it has the potential to be malignant. The current minimum age is only 26 days of birth,^[2]^ but huge literature claims that LCA is frequent in middle-aged people.^[3]^ The 10 patients in the current study comprised of middle-aged patients, and a majority of them had no subjective prior symptoms, but mostly due to accidental discovery during the examination. Some patients also had abdominal pain, abdominal distension, splenomegaly, hypersplenism, and other manifestations. Normal spleen cells did not express CD207 and Cyclin D1, while both LCA and lymphocytes showed CD207 (+) and Cyclin D1 (+),^[4]^ so it is speculated that its pathogenesis may be related to immune system abnormalities. Certain studies have suggested that LCA is linked with tumor necrosis factor-alpha, Crohn disease, and either Gaucher’s disease.^[5–9]^ Most studies also confirm that LCA is caused by various stimulating factors such as chronic infection or tumors.^[3]^ Approximately 30% of LCA patients are accompanied by various types of malignant tumors, most of which are visceral malignant tumors such as colorectal cancer, pancreatic cancer, and hepatocellular carcinoma. Urogenital system tumors, for example, renal cancer, testicular cancer, and ovarian cancer have also been the causes of the LCA. Lymphoma, myelodysplastic syndrome, and aplastic anemia are also quite common in the LCA patients.^[10]^ Keeping in view the smaller number of LCA currently reported, it is difficult to obtain solid evidence to clarify the pathogenesis, and the exploration of the pathogenesis still needs further cases and in-depth studies.

### 4.2. Imaging characteristics of LCA

Ultrasonography is superior to many of the related known methods for LCA.^[11]^ Ultrasonography shows nodular masses with uneven echo and small blood flow signals from the tumors. However, ultrasound is susceptible to the interference of the operator level and intestinal gas, so there is little accuracy in the diagnosis.

At present, CT has obvious advantages for examination of the character, blood supply, and the relationship between spleen tumor and the surrounding organs, and hence highly reliable tool for the diagnosis of LCA. In a plain CT scan, the density of the tumor was similar to that of a normal spleen, and the boundary was much more obvious. During the arterial phase of the enhanced scan tumors get clearer, manifested as single or multiple circular nodules with uneven mild enhancement, and the peripheral enhancement of the tumor remains unobvious. Contrast agents continued to fill in the portal phase, and the enhancement in the venous phase became balanced, and the enhancement degree of the tumor mimics normal spleen tissue. The number of lesions on enhanced CT showed a dynamic change process of “less-more-less.” The reason was that the nodules with equal density or signal intensity were not displayed in the plain scan, and the number of lesions remained small. In the arterial phase, the nodules with equal density or signal intensity could be clearly displayed under the contrast of the spleen with obvious enhancement, and the number of lesions appeared to be increased. Some lesions in the venous stage and delayed stage were of enhanced similar density. Therefore, the number of lesions decreases compared with the arterial stage.

During the MRI examination, LCA displayed an iso-low signal on T1WI and a high signal on T2WI. A small low-signal shadow was visible within the lesion, presenting a “freckle-like” appearance. This appearance was dependent on expression levels of hemosiderin stored in the littoral cells, which in turn had an association with the paramagnetic effect of iron. After contrast enhancement, the enhancement pattern of the lesions was similar to that of CT, revealing slow, progressive, and concentric enhancement.

### 4.3. Pathological characteristics of LCA

It hard to diagnose LCA patients prior to surgery, and postoperative pathology is crucial to clarify it. Most of the gross specimens showed that the volume of the spleen increased to varying degrees, and the surface or section of the spleen showed nodular changes, depicting single or multiple nodules of variable sizes. It is also reported that the highest diameter of LCA nodules may reach up to 21 cm,^[12]^ and these appeared spongy or cystic in cut surface, gray-white, dark red, or black-brown in color, but rarely white. Most of the nodules had clear boundaries with the surrounding spleen tissue. Under optical microscopy, the splenic capsule tissue was proliferated and thickened. The tumors proliferated and expanded sinusoid cavities of different sizes, some of which had papillary processes. The tumor endothelial cells were generally a monolayer of cuboidal or columnar cells with large rounded or ovoid nuclei, and vacuolated but eosinophilic chromatin. Granular material formed by hemosiderin was seen in the cytoplasm, and hyaline bodies of different sizes were also seen. PAS staining was positive, and some of them were detached and free in the lacunae. The immunophenotype showed dual expression of both vascular endothelial cell markers (CD31, CD34, FVIII) and histiocytic cell markers (CD68).

### 4.4. Differential diagnosis

1. Littoral cell angiosarcoma: It has a high degree of malignancy, rapid progression, often infiltrates surrounding tissues and organs, and the postoperative recurrence and metastasis appear so quickly with poor prognosis. Pathological histological examinations showed that blood vessels were anastomosed to each other, and endothelial cells showed nest-like or flake-like hyperplasia, often accompanied by hemorrhage and necrosis. Littoral cell angiosarcma is homologous to LCA. The IHC identifies the expression levels of biomarkers of endothelial and tissue cells, which are easy to get confused. The difference from LCA is that the sarcoma is positive for CD8 and CD34, while negative for CD21 and FVIII antigen, and high ki-67 proliferation index.
2. Splenic hemangioma: Splenic hemangioma is one of the frequent benign tumors of the spleen, which is divided into cavernous hemangioma and capillary hemangioma, without any specific general symptoms. There is rare part of the performance of the left upper abdominal pain or tumor rupture caused by bleeding. At the microscopic level, it showed vascular lumens of variable sizes, lined with endothelial cells, and only expressed endothelial cell characteristics such as CD31, CD34, and VIII factor-related antigen positive but did not express tissue cell antigen (CD68).
3. Splenic angiosarcoma: The incidence of splenic angiosarcoma is extremely low, and the time of onset is mostly about 50 to 60 years of age. The disease progresses quickly and the degree of malignancy is much higher and is accompanied by liver, lung, and other organ metastasis and regional lymph nodal metastasis. Its half-year survival rate is <20% and the clinical manifestations are not typical, so it is mainly relying on imaging diagnosis. Ultrasonography shows solid or mixed echo and a low echo halo can be seen around the tissues. CT plain scan showed splenomegaly with uneven density and borderline indistinct mass, which could be elaborated during the enhanced scan. During the MRI scan, the lesions in the spleen showed patchy long T1 and short T2 signals. The DWI sequence primarily showed low signals, and a few showed slightly higher signals. The enhancement of the lesions in the spleen parenchyma was not obvious. In histopathological examinations, splenic angiosarcoma is composed of an anastomosing cavernous vascular network. Tumor cells grow in nests or papillary along the vascular wall. The IHC characteristics are similar to LCA, and the important identification points are that the sarcoma endothelial cells are obviously atypia, mitotic and necrosis is frequent.
4. Splenic lymphangioma: Splenic lymphangioma is a benign tumor, frequent in young and middle-aged people, with atypical clinical symptoms. CT showed multiple low-density lesions of variable sizes. If the tumor was complicated with bleeding or infection, it could be manifested as high density or mixed density, and there could be calcification in the focal area. The enhancement was not obvious on the enhanced CT scan. A large number of thin-walled cavities were found in histological examinations, lined with a single layer of endothelial cells. Endothelial cells of lymphangioma could be accompanied by papillary hyperplasia. IHC of splenic lymphangioma showed positive CD31, CD34, and FVIII, whereas D2-40 is a marker that shows lymphatic endothelial cells and helps in differential diagnosis.
5. Splenic hamartoma: Splenic hamartoma is frequent in middle-aged people, with atypical clinical symptoms. The majority of them were single masses, and a few could be manifested as multiple masses without obvious capsules. CT showed isodensity or slightly low-density shadow, and few with mixed-density shadow. Enhanced scanning showed progressive enhancement over time. The microscopic composition was similar to that of a normal spleen, mainly red pulp. Abundant blood flow in the sinus cavity was seen, whereas lymphocytes were focally gathered, and splenic cord components with fibrous hyperplasia were also observed. IHC phenotypes CD8 were positive, while CD21 and CD68 remained negative.

### 4.5. Treatment and prognosis

LCA is mostly manifested as the biological behavior of benign tumors, but it is difficult to distinguish benign LCA from sinus littoral cell angiosarcoma. Therefore, surgical treatment should be the first choice for LCA and a clear diagnosis can be made. At present, laparoscopic splenectomy has become the preferred treatment option for LCA. For single tumors with limited lesions, a partial splenectomy can be performed after adequate evaluation.^[13]^ Compared with traditional open surgery, laparoscopic surgery has certain superiorities over other available options. In this group, 3 patients underwent open surgery, 7 patients underwent laparoscopic surgery, and 2 of them with laparoscopic partial splenectomy. Patients treated with laparoscopic surgery can get out of bed and have liquid diet on the following day post-surgery, with a shorter hospitalization period and quick recovery. Certain studies also suggested that open surgery with a spleen diameter exceeding 20 cm is safe, while Takayoshi and colleagues reported a case of LCA with liver metastasis 10 years after splenectomy, and used a chemotherapy regimen containing etoposide and paclitaxel for treatment, and achieved satisfactory therapeutic effect.^[14]^

All 10 patients inclusive of the study have been kept under follow-up by early of September, 2023 comprising the longest follow-up period was 12 years and 10 months. No recurrence of LCA and or other metastatic tumors was seen in these patients. Although the prognosis of LCA is generally better post-surgery, in a few cases certain other organ metastasis have also been seen. Therefore, it is recommended to follow up with patients after LCA for a long time with proper monitoring using multiple system indicators.

## 5. Conclusion

Due to the limited number of LCA cases, the current clinical understanding is relatively insufficient to define a solid treatment regimen, and diagnosis. With increased clinical cases along with the utilization of advanced techniques, the etiology, clinical characteristics, and biological behavior of LCA can be further explained. This will provide much facilitation in the future for quick and early diagnosis having string therapeutic regimens.

## Acknowledgments

The authors would like to thank the staff of the Department of general surgery “Qiang Yan” for providing clinical advice during the course of treatment.

## Author contributions

**Data curation:** Huaping Shen, Pengtao Song, Jian He.

**Formal analysis:** Yingjie Zhu, Yang Shen, Yang Huang.

**Investigation:** Jiajie Zhong.

**Project administration:** Xiaochang Wu.

**Resources:** Huaping Shen, Pengtao Song, Jian He, Shiyu Zhou, Xiaochang Wu.

**Validation:** Huaping Shen, Yingjie Zhu, Yang Shen.

**Writing – original draft:** Huaping Shen, Yingjie Zhu, Yang Shen.

**Writing – review & editing:** Huaping Shen, Shiyu Zhou, Xiaochang Wu.
